# Barriers to uptake of referral eye care services among the elderly in residential care: the Hyderabad Ocular Morbidity in Elderly Study (HOMES)

**DOI:** 10.1136/bjophthalmol-2021-320534

**Published:** 2022-04-01

**Authors:** Srinivas Marmamula, Thirupathi Reddy Kumbham, Satya Brahmanandam Modepalli, Subhabrata Chakrabarti, Jill Elizabeth Keeffe

**Affiliations:** 1 Brien Holden Institute of Optometry and Vision Science, L V Prasad Eye Institute, Hyderabad, India; 2 School of Optometry and Vision Science, University of New South Wales, Sydney, New South Wales, Australia; 3 Allen Foster Community Eye Health Research Centre, Gullapalli Pratibha Rao International Centre for Advancement of Rural Eye care, L V Prasad Eye Institute, Hyderabad, India; 4 Department of Biotechnology / Wellcome Trust India Alliance, L V Prasad Eye Institute, Hyderabad, India; 5 Prof. Brien Holden Eye Research Centre, L V Prasad Eye Institute, Hyderabad, India

**Keywords:** Epidemiology, Public health, Vision

## Abstract

**Background:**

To report on the barriers to uptake of eye care services after referral in the elderly in ‘homes for the aged’ in Hyderabad, India.

**Methods:**

Individuals aged ≥60 years were recruited from 41 ‘homes for the aged’ and were examined in the ‘make-shift’ clinics in homes. All participants who had vision impairment or needed further eye examination other than spectacles were referred to the higher centres for ‘free services’. Three months after the referral, the participants were interviewed and asked about the uptake of services, and their reasons for not attending.

**Results:**

In all, 731/1182 (61.8%) participants were referred of which 375 (49.9%) attended. In multiple logistic regression, participants aged ≥80 years were less likely to utilise the services (OR 0.60; 95% CI 0.39 to 0.03). Similarly, the participants living in free homes (OR 3.53; 95% CI 2.15 to 5.79) and subsidised homes (OR 2.24: 95% CI 1.55 to 3.23) and those independently mobile had higher odds for uptake of services (OR 5.74; 95% CI 3.31 to 10.51). The major reasons for not availing the referral services were ‘lack of felt need’ reported by 136 (45.4%) participants followed by other health issues in 100 (33.4%) participants and non-consenting family members in 49 (16.4%) participants. In all, 14 (4.7%) participants gave other reasons.

**Conclusions:**

The uptake of eye care services in the elderly in residential care remains poor despite the provision of services for free. Lack of felt need for services is the main reason for non-compliance to the referral for care. Counselling on the benefit of interventions could potentially improve referral compliance in this population.

**Key messages**

**What is already known on this topic**

- Though the burden of vision impairment among elderly in residential care is known, there is limited information on barriers to uptake of referral services. Understanding the barriers will help develop eye health programmes with improved uptake of services.

**What this study adds**

- About half of the elderly do not comply for referral advice. Lack of felt need and systemic health condition are the important reasons poor compliance to referral services.

**How this study might affect research, practice or policy**

- The study highlights the need to develop strategies to address the barriers to uptake of services in the elderly in residential care in India.

## Introduction

Globally, vision loss affects over a billion people.^1^ The majority of the conditions leading to vision loss (~80%) are attributable to cataract and refractive errors and can be addressed with simple interventions in the form of providing cataract surgery and spectacles, respectively.^2^ Vision loss is also associated with mortality^3 4^ and adversely impacts the quality of life and well-being of the elderly.^5 6^ In the Indian scenario, 8% of the population is aged ≥60 years (elderly), which is expected to rise to 20% by the year 2050.^7^ This translates to 195 million elderly people by the year 2030 and 324 million by the year 2050.^7^ Due to an increase in senescence, contributed by changes in lifestyle and rapid urbanisation, the numbers of elderly either living alone or with their spouses is on the rise. This has also led to an increase in the number of homes for the aged in India, which is currently estimated to be around 1000 homes housing over 100 000 residents in them. This number is likely to increase in time.^8^

Vision impairment (VI) is disproportionately high among the elderly population in the communities.^9^ It is even higher among the elderly in residential care in India.^10^

To address vision loss in the elderly, a comprehensive strategy is essential. This strategy includes identification of the elderly with vision loss, providing them with spectacles, and further referral of complex cases for medical/surgical intervention. The uptake of appropriate and timely eye care by people with vision loss has been a major cause of concern across all age groups.^11 12^ Likewise, there are perceived barriers that limit the elderly from seeking higher levels of eye care following primary referral.^13^ Unfortunately, very limited data are available on barriers to the uptake of eye care services among the elderly both in the population and among those living in residential care. Understanding the barriers that prevent elderly from seeking eye care is essential to develop holistic strategies to address VI and resolve other eye health issues. These strategies in turn could contribute towards overall health and well-being of the elderly.

Towards this, the HOMES (Hyderabad Ocular Morbidity in Elderly Study) was conducted among the elderly in residential care (homes for the aged centres) in Southern India with the primary aim to assess the prevalence, causes and risk factors for VI in this population.^14^ We had earlier reported that over 30% of the elderly in the residential care had VI and it was avoidable in 90% of them.^10^ We had also provided spectacles for all the elderly and had referred them for further interventions where it was needed on a complimentary basis. Herein, we report on barriers to uptake of referral services among this elderly population in residential care.

## Materials and methods

Written informed consent was obtained from all the participants and the study was conducted in accordance with the tenets of the Declaration of Helsinki. The study was carried out in homes for the aged centres in the city of Hyderabad and its adjoining regions in the south Indian state of Telangana.^14^ Only participants aged ≥60 years at the time of enumeration and residing at one of these homes for at least a month, were included in the study.

### Examination procedure

The HOMES eye examination protocol has been described in our earlier publications.^10 14^ In brief, the data on personal and demographic characteristics including age, gender, level of education and self-reported systemic conditions such as diabetes and/or hypertension, were collected using precoded questionnaires. The physical mobility status of the participants was classified as ‘independently mobile’, ‘mobile with assistance’ or ‘immobile/bedridden’. The homes for the aged were classified as (1) Private homes, where the individuals or their kin paid a monthly or annual user fee, (2) Aided/partially subsidised homes, where the individuals or their kin paid a part of the user fee, and the rest was met by philanthropic support or other funding sources and (3) Free homes, where individuals did not have to pay any user fee as these homes were entirely supported by external funding sources. Comprehensive eye examinations were done that included visual acuity (VA) assessment, refraction, slit-lamp examination, intraocular pressure measurement and fundus imaging. Further details are provided in a previous publications.^10 14^

### Definitions

VI was defined as presenting VA worse than 6/18 in the better eye. This was further subclassified into blindness (worse than 3/60), severe VI (worse than 6/60–3/60) and moderate VI (worse than 6/18–6/60).

### Services

Spectacles were directly provided at the homes for all the participants with uncorrected refractive errors and presbyopia. The elderly with VI due to other causes such as cataract and/or those who needed additional care were referred to the L V Prasad Eye Institute for intervention. This facility is located within 1-hour drive from the homes. A referral letter was provided to the participants and all services are facilitated by dedicated personnel at the institute. All services were provided at ‘no cost’ to the participants.

### Interviews

Trained interviewers (personnel with a master’s degrees in social work and qualitative research) visited the homes, at least 3 months after the referral and interviewed participants. The interviews were carried out in the local language (Telugu or Hindi) in a calm and comfortable environment in a private setting. The interviewers are familiar to the participants and the settings as they were involved in the early phases of data collection for the study.

An open question on ‘Why have you not utilized the referral services?’ was asked to the participants and their responses were recorded on the response sheet verbatim. The corresponding text was later reviewed by the principal investigator (SM) and were classified into the following categories for analysis. These included (1) No felt need (included responses such as not keen for services, can manage with existing vision, not interested at this age), (2) No family consent (included where participants are interested to visit the institute for services but they either had no approval from their family members or they were not willing to take them to the institute), (3) Other health issues (such as hypertension and other serious health issues preventing the hospital visits) and (4) Other reasons (included the remaining responses other that mentioned above). These categories are not prespecified but based on the responses given by the participants.

### Data analysis

Data analysis was conducted using Stata Statistical Software for Windows, V.14 (StataCorp). Descriptive analyses were conducted. Multiple logistic regression analysis was done to assess the predictors for the uptake of eye care services and presented as adjusted OR with 95% CIs.

## Results

### Study participants

**Figure 1:**
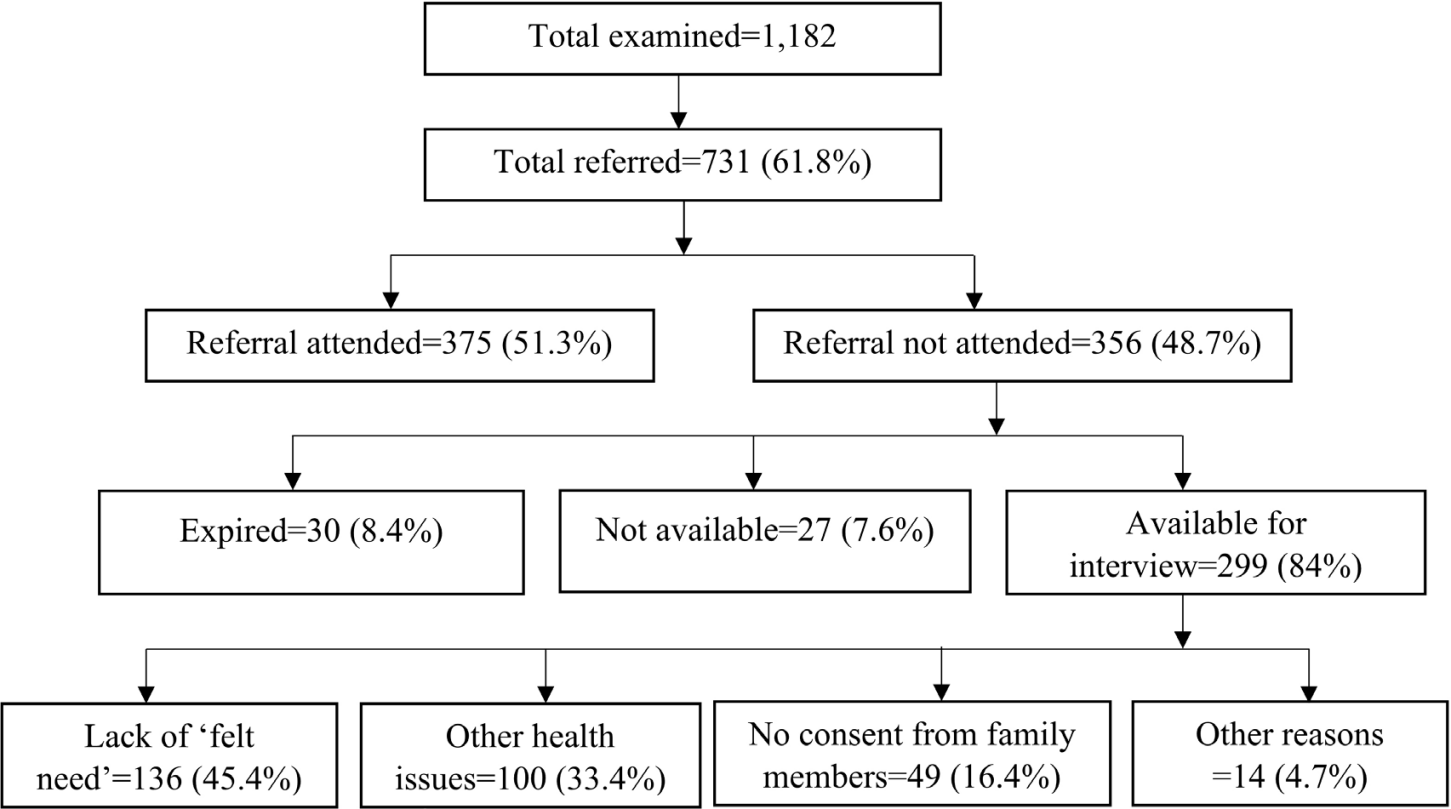
Flow chart showing the availability of participants and reasons for non-compliance to referral advice.

Overall, 1182 participants were examined from 41 homes for the aged, of whom 731 (61.8%) participants were referred for higher levels of care. The mean age (SD) of the participants was 75.9±9.0 years (range: 60–108 years); 65.8% (n=481) were women and 25% (n=183) had no formal education. Additionally, 18.2% (n=133) of the participants were from free homes and 12.2% (n=89) were bedridden or immobile. Among the participants referred, 45.1% (n=330) had VI in their better eye. In terms of the type of referral, 47.3% (n=346) were referred for cataract surgery, 12.9% (n=94) were referred for YAG (Neodymium-doped yttrium aluminum garnet) laser capsulotomy for posterior capsular opacification (non-surgery referrals) and the remaining 39.8% (n=291) were referred were consultations for other conditions (non-surgery referrals). These included retinal disorders (n=192; 26.8%) and glaucoma (n=62; 8.5%). Among those referred, around 50% (n=375) had attended the services (figure 1).

On univariable analysis, the uptake of referral services was highest (59.1%) among those who were independently mobile, compared with those who needed assistance or were bedridden (p<0.01). The uptake of services was significantly higher (p=0.048) in those with no VI in the better eye compared with those had VI (54.6% vs 47.3; p=0.048). On the other hand, there were no differences the uptake of services among those referred for cataract surgery (54.6%) and other services (50.2%) (table 1).

**Table 1:** Characteristics of the participants who were referred and availed services (n=731)

	Total—referred	Referrals not attended n (%)	Referrals attended n (%)	P value
Age group (years)				<0.001
60–69	181	74 (40.9)	107 (59.1)	
70–79	277	116 (41.9)	161 (58.1)	
80 and above	273	166 (60.8)	107 (39.2)	
Gender				0.172
Male	250	113 (45.2)	137 (54.8)	
Female	481	243 (50.5)	238 (49.5)	
Education level				0.65
No education	183	86 (47.0)	97 (53)	
School education	431	216 (50.1)	215 (49.9)	
Higher education	117	54 (46.2)	63 (53.8)	
Type of home				<0.001
Free	133	43 (32.3)	90 (67.7)	
Aided/partially subsidised	289	120 (41.5)	169 (58.5)	
Private	309	193 (62.5)	116 (37.5)	
Diabetes				0.281
No	535	267 (49.9)	268 (50.1)	
Yes	196	89 (45.4)	107 (54.6)	
Hypertension				0.405
No	313	158 (50.5)	155 (49.5)	
Yes	418	198 (47.4)	220 (52.6)	
Mobility score				<0.001
Immobile/bedridden	89	73 (82.0)	16 (18)	
Mobile with support	253	136 (53.8)	117 (46.2)	
Independently mobile	389	147 (37.8)	242 (62.2)	
Visual impairment in the better eye				0.048
No	401	182 (45.4)	219 (54.6)	
Yes	330	174 (52.7)	156 (47.3)	
Total	731	356 (48.7)	375 (51.3)	

### Predictors of referral uptake

Multiple logistic regression analysis indicated that those aged 80 years and older were less likely to attend the referral services (OR 0.60; 95% CI 0.39 to 0.93) compared with the younger groups. The referral uptake was higher among participants residing in aided/partially subsidised homes (OR 2.24; 95% CI 1.55 to 3.23) and free homes (OR 3.53; 95% CI 2.15 to 5.79) compared with those residing in private homes. Similarly, the uptake of services was higher in subjects who were independently mobile (OR 5.74; 95% CI 3.13 to 10.51) or those mobile with assistance (OR 3.65; 95% CI 1.96 to 6.80) compared with those who were immobile/bedridden. Gender (p=0.05), level of education (p>0.05), self-report of diabetes (p=0.44) and hypertension (p=0.133) and were not associated with referral uptake. Though VI was significant on univariable analysis, it was not associated with the uptake of services (table 2).

**Table 2:** Associations of referral uptake with sociodemographic and clinical characteristics based on multiple logistic regression analysis (n=731)

	OR (95 % CI)*†	P value
Age group (years)		
60–69	Reference	
70–79	1.07 (0.71 to 1.62)	0.739
80 and above	0.6 (0.39 to 0.93)	0.023
Gender		
Male	Reference	
Female	0.70 (0.49 to 1.00)	0.051
Education level		
Higher education	Reference	
School education	0.70 (0.44 to 1.14)	0.155
No education	0.84 (0.47 to 1.50)	0.555
Home type		
Private	Reference	
Aided/partially subsidised	2.24 (1.55 to 3.23)	<0.01
Free	3.53 (2.15 to 5.79)	<0.01
Mobility score		
Immobile/bedridden	Reference	
Mobile with support	3.65 (1.96 to 6.80)	<0.01
Independently mobile	5.74 (3.13 to 10.51)	<0.01
Visual impairment in the eye		
No	Reference	
Yes	0.82 (0.58 to 1.14)	0.238
Diabetes		
No	Reference	
Yes	1.16 (0.79 to 1.7)	0.444
Hypertension		
No	Reference	
Yes	1.3 (0.92 to 1.82)	0.133

*Hosmer-Lemeshow test for goodness of fit for the regression model, p=0.64.

†Mean-variance inflation factor for the multiple logistic regression model=1.28.

### Reasons for non-compliance with referrals

**Figure 2:**
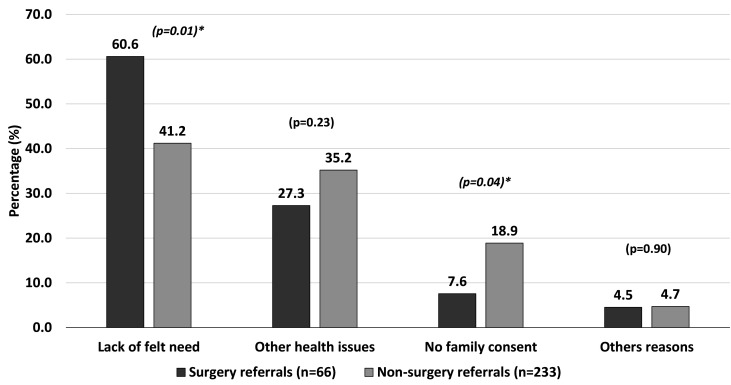
Reasons for non-compliance to referrals stratified by type of referral. (*statistically significant)

Among the referred participants who did not attend the services (n=356), 8.4% (n=30) were deceased and 7.6% (n=27) had moved out of the home. The major reasons for not availing the referral services for the remaining 299 (84%) participants were ‘lack of felt need’ reported by 136 (45.4%) participants followed by other health issues in 100 (33.4%) participants and non-consenting family members in 49 (16.4%) participants. In all, 14 (4.7%) participants gave other reasons. The reasons for non-compliance differed by category of referral. ‘Lack of felt need’ was more among those referred for surgery compared with non-surgical referrals (60.6% vs 41.2%; p=0.01). The reasons for referral non-compliance stratified by type of referral are shown in figure 2.

## Discussion

Half of the elderly in residential care failed to comply with the referral despite the availability of services at no-cost to them. This is a matter of grave concern for programme planners and eye health service providers. It also suggested the need for a comprehensive approach to ‘close the loop’ to ensure that eye care finally reaches those in need. Lack of felt need was the foremost reason for non-compliance to referral for eye care services. This could also be attributed to several reasons such as lifestyle and visual demands in the home care settings. Another possible reason could be sheer disinterest, loss of hope, or a sense of tiredness in the latter part of life due to more precipitating events that led to their stay at home. The loss of one’s familiar social network along with the perception of being forced to stay at a home may add to their feel-bad fatalism. Earlier studies have demonstrated the importance of a sense of belongingness to an existing social network for the well-being of the elderly.^15 16^ Additionally, there was also a perceived element of risk and/or felt hardship among those who were advised for cataract surgery as opposed to the benefits of undergoing surgery.

Multimorbidity and disability are common among the elderly.^17 18^ Though there are limited data on the prevalence of multimorbidity in the elderly in residential care, the published literature on the elderly in the community suggests a high burden. We reported a higher prevalence of vision loss among the elderly with mobility issues in the homes.^10^ Poor mobility imposes greater dependency, resulting in the poor uptake of services. Lack of consent of family members for seeking care is a serious matter and highlights the intricate dependence of the elderly on significant others. Such denial of care to the elderly constitute ageism, which is very common in India but seldom reported.^19^ Ageism refers to the concept of how people think, feel and act towards others, due to ageing factors.^19–21^ It is reported to have an adverse impact on the health and well-being of the elderly, as they face the brunt of this targeted behaviour, which is in turn ascribed to their advancing age and dependency on their kith and kin for basic needs.^20–22^

Older age and being care-dependent are factors associated with ageism.^20–22^ The elderly with vision loss in homes for the aged are particularly vulnerable to this phenomenon as they are directly dependent on the decision of the family member(s) to seek care. Unfortunately, this lack of support precludes a significant proportion of these participants from benefiting through clinical interventions and thereby degrading their quality of life. Also, the lack of felt need among the elderly may be attributed to ageism and disinterest in life due to several personal and social reasons.

We observed that the elderly in free homes were more likely to comply with referral advice as they were relatively more independent to take decisions related to their health issues. They are either devoid of family members or have been completely abandoned by them. In contrast, the family members of the elderly still tend to influence them individual on the decision to seek care in private homes. Their decision appears to be binding as they financially support the stay of the elderly at these homes. Thus, there was a clear trend showing high uptake of referral services by those residing in free homes, followed by those in aided/subsidised homes, while the least uptake of referral for services was observed in private homes. Possible reasons for denial of permissions to seek care could be perceived risk of complications of cataract surgery in advanced age which may adversely impact their lives. Other reasons include time commitment for post-operative care and follow-up visits. Interestingly, VI was not the driving force for their uptake of services, rather this could be attributed to a less demanding lifestyle and lesser range of visual needs for those living in homes for the aged.

Utilisation of eye care services in general and cataract surgeries, in particular, have been extensively documented in the Indian context.^12 23–25^ Most of these studies were based on primary referrals from the communities.^12 23–25^ Lack of escort, financial issues and more recently person-related issues like lack of felt need have been frequently reported.^12 23–25^ However, most of these studies were population-based and often included participants either in age groups of 40 years and older or those above 50 years. Direct comparison to these studies with our home-based study with older participants may not be justified.

### Implication for eye care service providers

This study highlights the intricate issues related to referral compliance by the elderly in residential care. While vision loss is a challenge in these elderly and existing barriers to compliance with the care regimen advised further compound the situation, a multipronged approach is needed to address this emerging challenge. There is a need for home-based care where specialty services could be provided using technological advancements such as teleophthalmology to minimise hospital visits. This is especially true for chronic conditions such as glaucoma and diabetic retinopathy which need periodic assessments. More than half of the elderly in our study were referred for non-surgical interventions and follow-up care but going forward these could be effectively addressed using advanced digital technology. Ageism that is prevalent in the elderly population can be addressed using targeted education programmes including the positive impact of good vision on their activities of daily living may help convert lack of felt need into perceived need for services.^26^

While cataract surgery is becoming more accessible in India based on the increasing cataract surgical rate (number of cataract surgeries per million population per year), other age-related ocular conditions are likely to increase in the future. Hence, there is a need to systematically address these changing trends leading to vision loss in the elderly in residential care. Also, understanding the perspective of caregivers and/or family members may be essential to address the barriers for uptake of services.

The prevalence of disabilities and non-communicable diseases increases exponentially with ageing. Given the success rate of cataract surgery with good visual outcomes, it is recommended that surgery be advised early in life before the onset of other systemic health conditions. In India, there is a general tendency to delay cataract surgery and wait for the cataract to mature before the person is ready to have the eye operated. At times, the waiting becomes too long and the decision for surgery becomes challenging which could sometimes be detrimental due to the onset of other systemic conditions in parallel, which further increases the dependency of the elderly. There appears to be a ‘Cataract Window’ or a window of opportunity where safe cataract surgery could be carried out based on the visual threshold, age, other systematic conditions and limited anesthesia-related complications. This needs to be explored further and is beyond the scope of the present work.

## Conclusion

This is the first study to report on the barriers to poor uptake of eye care services among the elderly living in residential care in India. Inclusion of a large number of homes and in-depth eye health assessment and tailored interviews are the major strengths of this study. The emerging theme of ‘lack of felt need’ and poor uptake of services in private homes require further research using more in-depth qualitative research methods. In addition, data on perspectives of care givers and services providers may provide insights for developing interventions to address the barriers.

With changing social structure and simultaneous increasing elderly population, the number of homes is likely to increase in India. It is time to design appropriate strategies and protocols so that the elderly residing in these homes do not needlessly suffer from the adversities of vision loss. Proper implementations of evidence-based technology-enabled care closer to homes for the elderly are mandated to achieve the ambitious goal of universal eye health.

## Data Availability

All data relevant to the study are included in the article or uploaded as online supplemental information. Not applicable.
